# Freeform Mirror Design for Novel Laser Warning Receivers and Laser Angle of Incidence Sensors

**DOI:** 10.3390/s20092569

**Published:** 2020-04-30

**Authors:** Jacek Wojtanowski, Marcin Jakubaszek, Marek Zygmunt

**Affiliations:** Institute of Optoelectronics, Military University of Technology, 00-908 Warsaw, Poland; marcin.jakubaszek@wat.edu.pl (M.J.); marek.zygmunt@wat.edu.pl (M.Z.)

**Keywords:** freeform mirror, angle of incidence measurement, AOI sensor, laser warning receiver, LWR, laser warning system, LWS, non-imaging optics, laser incidence angle sensing

## Abstract

In this paper, we present a novel configuration of an optical angle-of-incidence (AOI) sensor based on the application of a freeform mirror. The main challenge in designing this mirror was to provide a strictly linear transformation between AOI and the spatial position of the spot created on the linear detector array. Another two goals of this paper were to minimize stray light issues (improve the dynamic range) and create an intermediate focus and lateral shift in the detector position with respect to the plane of incidence. From an optical point of view, the designed mirror can thus be understood as the composition of three components: a high-numerical-aperture (NA) fully achromatic f-theta lens in one cross-section and a perfectly focusing lens, combined with a deviating prism in the second (orthogonal) cross-section. In comparison to the standard “shade” methods, the proposed approach allows a constant angular resolution to be maintained over the entire field of view. The mirror was designed on the basis of fundamental geometrical rules by numerically solving differential problems using an innovative scheme based on the minimization of the specific merit function. The proposed method was practically applied to design a freeform mirror for a 90°/120° field-of-view sensor, showing a satisfactory performance.

## 1. Introduction

The detection of incoming laser radiation is associated with an important and constantly expanding group of security, military, aircraft, and satellite safety systems [1]. Lasers that should be detected by laser warning receivers/systems (LWR/LWS) correspond to an extremely large family and, for this reason, show a very broad energy/power spectrum. Additionally, taking into account that the distance to a detected laser transmitter can vary, modern LWR/LWS systems are faced with the requirement for a high sensitivity (order of a single W/m^2^, delivered, for example, by state-of-the-art laser rangefinders) and, at the same time, an extremely large dynamic range (the use of powerful Q-switched lasers may be associated with hundreds of kW/m^2^). Finally, it should be mentioned that the electronic bandwidth of the current LWR/LWS system has to be broad due to the quasi-cw and pulsed regimes (order of nanosecond pulse widths) of most operational laser-based systems.

**Figure 1:**
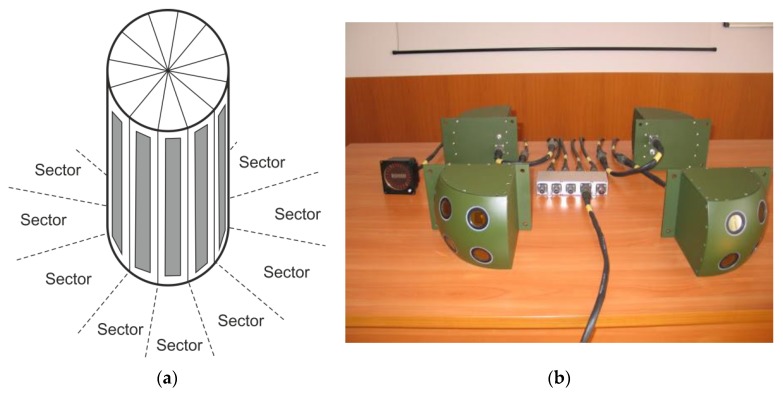
(**a**) Idea of a low-angular-resolution, multi-sector laser warning receiver (LWR) scheme. (**b**) Photo of laser warning system (LWS) “Procjon,” developed in our institute in the past (source: http://www.ioe.wat.edu.pl).

Nowadays, in most cases, laser warning systems, apart from detecting laser radiation, determine the direction from which a beam arrives [2], which is called the angle of incidence (AOI) measurement capability. This is vital, especially if an integrated countermeasure system is under consideration [3,4,5]. AOI determination allows for tracking the laser source and reacting accordingly. Depending on the required angular precision and total field of view, a variety of methods can be applied to measure AOI. In the case of coarse measurements (resolution over dozens of degrees) in a single plane (typically azimuth), it is sufficient to apply a few separate point detectors, oriented in respective directions and separated by mechanical blinds (Figure 1) [6]. Modern solutions, however, aim at a higher angular resolution possibly to allow for the effective and precise tracking of laser transmitters.

**Figure 2:**
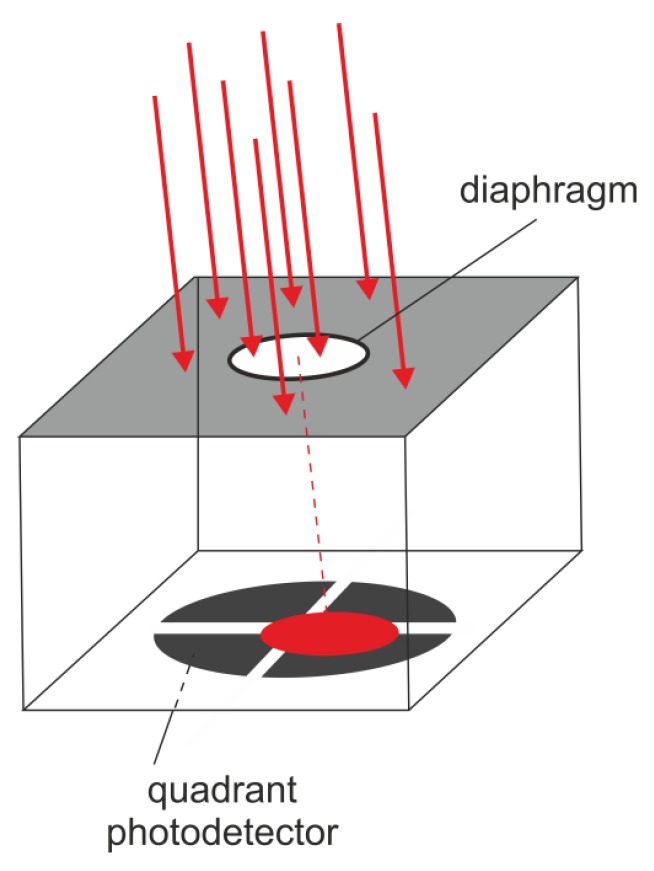
Idea of an LWR based on a quadrant photodetector.

**Figure 3:**
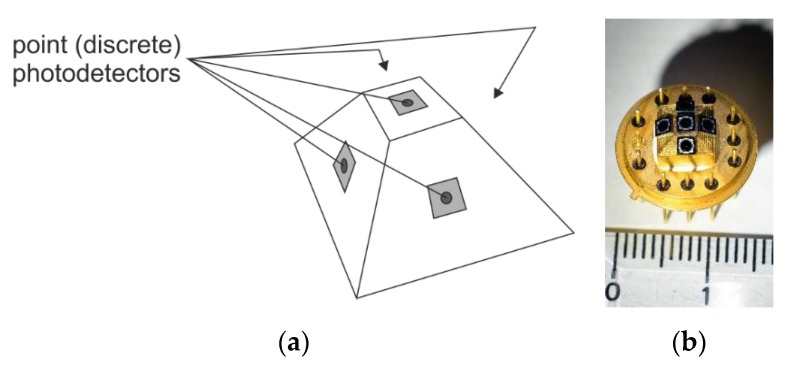
(**a**) Idea of an angle-of-incidence (AOI) sensor based on five discrete detectors bonded to a pyramidal geometry. (**b**) Photo of such a sensor developed by our team [8].

Another methodology deals with the application of quadrant photodetectors (Figure 2), or discrete point detectors, located on a selected geometry (a pyramidal shape is shown as an example in Figure 3). In this family of sensors, the ratios of received powers (and related photocurrents) allow for the evaluation of the angle of the incoming radiation [7].

In most cases, however, such solutions suffer from a limited field of view, low angular resolution, susceptibility to the background, and strong dependence on the beam irradiance distribution. A laser beam, even if initially perfectly Gaussian, after travelling hundreds of meters in the atmosphere, is strongly affected by refractive index spatial inhomogeneities (turbulence), which distort the wave front and produce irradiance inhomogeneities.

Thus, this kind of angular precision in an AOI sensor is limited by the atmospheric influence, which is strongly variable in time and depends on the measurement conditions. We experimentally observed a significantly better performance of the sensor presented in Figure 3b in windless winter weather, in comparison to a sunny summer day, over an asphalt road. Nevertheless, such solutions seem to be attractive for incoherent source tracking (sun positioning) and an LWR dedicated for a space environment (lack of atmosphere). 

**Figure 4:**
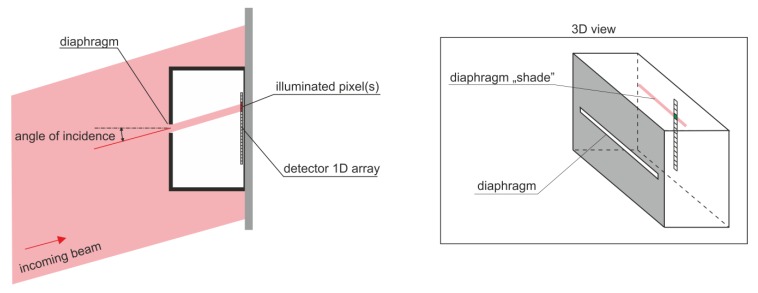
Idea of the “shade method”, commonly applied in most high-resolution AOI sensors.

Considering their requirement for a higher angular resolution of AOI sensors (order of single degrees), array detectors (1D or 2D matrix, composed of individual point detectors) are typically used in the so-called “shade method” configurations (Figure 4) [9,10]. A photodetector is arranged with some diaphragm in such a way that the incoming laser radiation, depending on the angle of incidence, illuminates selected pixels within the array. Thus, the activated pixel locations clearly correspond to the AOI or, more precisely, its tangent, the spatial coordinate of the activated pixel ~tan (AOI). The inconvenience of such an approach becomes a nuisance when a large field of view is considered. This results from the tangent function’s non-linearity and the non-uniform angular resolution within the field of view. 

For example, in the case of a 45° half field of view, the angular resolution in the center and at the edges differs twice. Apart from the resolution’s non-uniformity, the common “shade method” configurations suffer from stray-light issues. This results primarily from the significant net area of the diaphragm, which allows a lot of parasitic light into the sensor chamber. Considering the high-power lasers or short distances of detection, this corrupts the sensor operation. 

**Figure 5:**
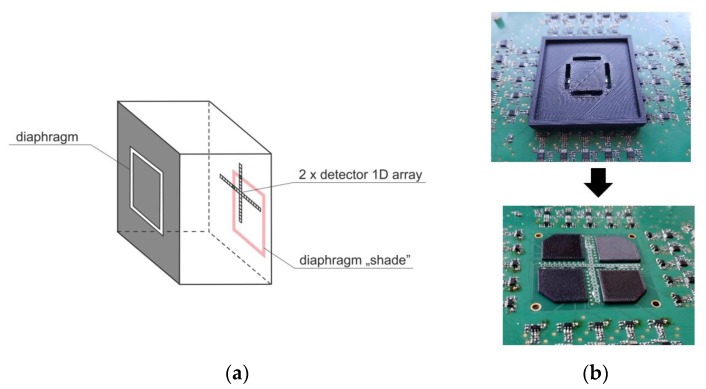
(**a**) Idea of a 2D configuration determined by the “shade method” for an AOI sensor. (**b**) Photos of such a sensor developed by our team (top: Sensor with the diaphragm, bottom: Diaphragm removed and 41 discrete PIN photodetectors arranged in a cross-plan).

Similar issues occur if 2D configurations of this method are taken into account (the example in Figure 5). Such setups are used if two angles of incidence need to be determined (for example, both azimuth and elevation).

The AOI sensor presented in Figure 5b, apart from the strong dependence of the angular resolution on the AOI, was strongly limited by stray light. While the internal surfaces were covered with a dedicated low-reflectivity coating, an irradiance exceeding 10^4^ Wm^−2^ activated all pixels, irrespective of the AOI. This makes such sensors useless, if high-peak-power pulsed lasers are considered (for example, Nd:YAG), even if measured at a distance of several kilometers.

**Figure 6:**
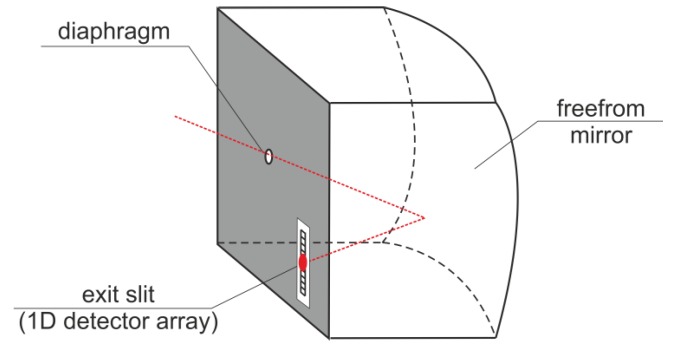
The proposed idea of a freeform mirror application in an AOI sensor.

To overcome the discussed drawbacks of most common current AOI sensor configurations, in this paper, we describe the novel configuration based on a 1D linear detector, cooperating with a freeform mirror and a small pinhole diaphragm (Figure 6).

The fundamental goal of the freeform mirror application in the proposed sensor was to ensure a constant angular resolution over the entire field of view. This was achieved by enforcing linearity between the spatial coordinate of the activated pixel and the AOI (not its tangent). 

Secondly, due to the considered 1D AOI sensor setup, the mirror was designed to direct the narrow pencil of light created by the small pinhole diaphragm exactly to the desired pixel within the detector 1D array, in such a way that this pixel number depends on the measured angle (for example, in Figure 6, it is, conceptually, elevation) and is independent of the orthogonal angle (following the example in Figure 6, it is independent of azimuth). 

**Figure 7:**
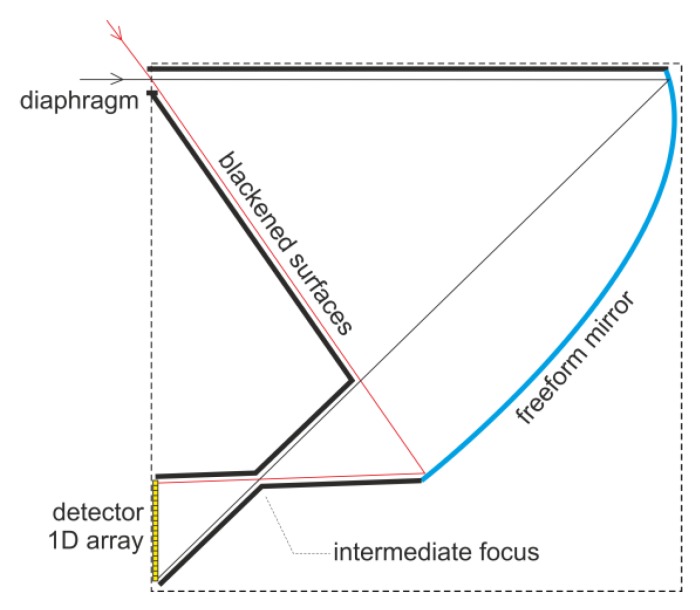
Configuration with an intermediate focus on minimizing stray light.

Finally, in order to minimize stray light, it was desirable to design a light-focusing geometry with an intermediate focus positioned somewhere within a chamber (Figure 7). Such a beam waste enables the creation of a mechanical bottleneck effect in the chamber, which will effectively block unwanted stray light. 

Stray light in LWR sensors is created by the ambient background, which enters the chamber through a diaphragm and increases noise in all detectors. Even more corrupting is a stray light factor produced in the case of powerful laser measurements. The beam scatters on the diaphragm edges, and especially if the diaphragm has a significant net area, a lot of light enters the chamber, where it undergoes multiple reflections. Apart from the diaphragm area and chamber geometry, the magnitude of the stray light impact depends on the chamber’s internal blackening quality. Each AOI sensor has a certain maximum incident power density, beyond which stray light activates the pixels, which should not be activated, leading to completely wrong AOI calculation results. In this way, the stray light issue limits the upper level of the sensor’s dynamic range (the lower level depends on the inherent sensitivity of the detectors).

## 2. Materials and Methods

In the following discussion, an optical design of a freeform mirror devoted to our novel 1D AOI sensor configuration is presented. Here, we consider the AOI elevation measurement. Obviously, the designed component can be easily transformed into an azimuth AOI sensor, simply by turning it 90°. Additionally, if both azimuth and elevation AOIs (2D LWR configuration) are needed, one has to apply two 1D LWRs oriented orthogonally.

**Figure 8:**
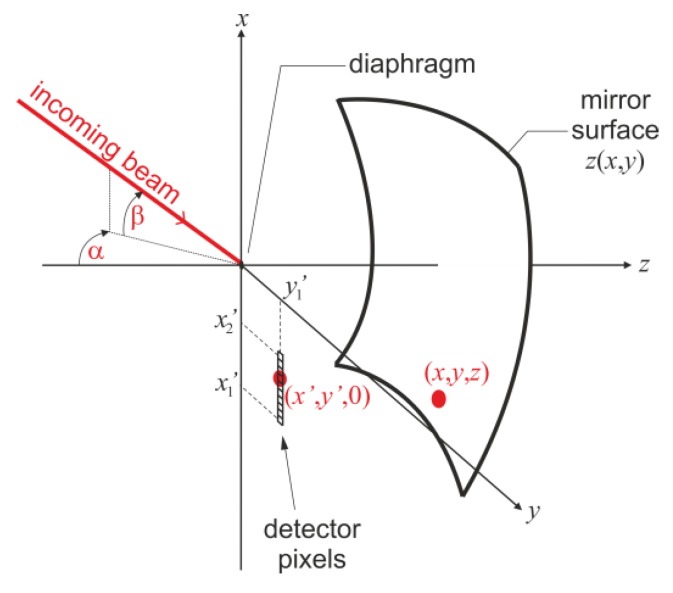
Geometry of the optical problem.

Next, we consider the coordinate system, as presented in Figure 8. The diaphragm is located at the origin (0, 0, 0), the freeform mirror surface is denoted as *z*(*x*, *y*), and the 1D linear detector array is located on the *xy*-plane and is parallel to the *x*-axis.

According to the previous discussion, the task of the mirror is to direct light rays to the appropriate pixel, which is determined by the incoming beam elevation angle (denoted as *β*), irrespective of the azimuth angle (denoted as *α*). 

Now, we derive the differential equation, which describes the mirror surface performing such a task. The incoming ray direction, represented by the (*α*, *β*) coordinates (Figure 8), can be considered as a line joining the diaphragm at (0,0,0) and a point on the mirror at (*x*, *y*, *z*), so the direction unit vector is as follows:

(1) $${\hat{s}}_{1}=\frac{\left[x,y,z\right]}{\sqrt{x^{2}+y^{2}+z^{2}}}$$

The angle of interest (*β*) and the second angle (*α*) clearly define the constraints on the coordinates of the point on the mirror that the beam will be reflected from, namely:

(2) $$\beta =\mathrm{atan}\left(\frac{-x}{\sqrt{y^{2}+z^{2}}}\right)$$

(3) $$\alpha =\mathrm{atan}\left(\frac{y}{z}\right)$$

After the reflection, the ray should propagate to a point (*x*’, *y*’, 0) in the detector plane (*xy*-plane). Thus, the reflected ray directional unit vector has the following general form:

(4) $${\hat{s}}_{2}=\frac{\left[x^{\prime }-x,\;\;\;\;y^{\prime }-y,\;\;-z\right]}{\sqrt{{\left(x^{\prime }-x\right)}^{2}+{\left(y^{\prime }-y\right)}^{2}+z^{2}}}$$

Concerning the desired property of the linear relationship between the angle *β* and detection pixel coordinate *x*’, the following condition should be met:

(5) $$\{\begin{array}{c} x^{\prime }\left(\beta \right)=x_{1}^{\prime }+\frac{\beta }{\beta_{max}}\left(x_{1}^{\prime }-x_{2}^{\prime }\right)\\ y^{\prime }=y\prime_{1}=const \end{array}$$

where *x*_1_’ and *x*_2_’ determine the start point and endpoint (see Figure 8) of the linear detector array, respectively (the difference between the two is equal to the length of the array), and *β*_max_ is the maximum elevation angle. Please note that according to the convention presented in Figure 8, (*x*_1_’ < 0, *β* > 0), the “+” sign in Equation (5) allows the intermediate focus to be obtained. Considering Equations(2) and Equation (5):

(6) $$x^{\prime }\left(x,y,z\right)=x_{1}^{\prime }+\frac{\mathrm{atan}\left(\frac{-x}{\sqrt{y^{2}+z^{2}}}\right)}{\beta_{max}}\left(x_{1}^{\prime }-x_{2}^{\prime }\right)$$

The difference between the two unit vectors (associated with the mirror local normal vector direction [11]) can easily be obtained from Equations (1) and (4):

(7) $${\hat{s}}_{1}-{\hat{s}}_{2}=\left[\begin{array}{c} \frac{x}{\sqrt{x^{2}+y^{2}+z{\left(x,y\right)}^{2}}}-\frac{x^{\prime }\left(x,z\right)-x}{\sqrt{{\left(x^{\prime }\left(x,y,z\right)-x\right)}^{2}+{\left(y^{\prime }-y\right)}^{2}+{\left(z\left(x,y\right)\right)}^{2}}},\\ \frac{y}{\sqrt{x^{2}+y^{2}+z{\left(x,y\right)}^{2}}}-\frac{y^{\prime }-y}{\sqrt{{\left(x^{\prime }\left(x,y,z\right)-x\right)}^{2}+{\left(y^{\prime }-y\right)}^{2}+{\left(z\left(x,y\right)\right)}^{2}}},\\ \frac{z}{\sqrt{x^{2}+y^{2}+z{\left(x,y\right)}^{2}}}+\frac{z}{\sqrt{{\left(x^{\prime }\left(x,y,z\right)-x\right)}^{2}+{\left(y^{\prime }-y\right)}^{2}+{\left(z\left(x,y\right)\right)}^{2}}} \end{array}\right]$$

Transforming the above formula into the **N** = (*p*, *q*, –1) form and taking into account Equations (2), (3) and (7), we obtain partial derivatives, *p* = ∂*z*/∂*x*, *q* = ∂*z*/∂*y*, which allows two partial differential equations governing the mirror surface to be provided:

(8) $$\frac{\partial z}{\partial x}=\frac{\left[x_{1}^{\prime }+\frac{\mathrm{atan}\left(\frac{-x}{\sqrt{y^{2}+z^{2}}}\right)}{\beta_{max}}\left(x_{1}^{\prime }-x_{2}^{\prime }\right)-x\right]S_{1}-xS_{2}}{z\left(S_{1}+S_{2}\right)}$$

(9) $$\frac{\partial z}{\partial y}=\frac{(y_{1}{}^{\prime }-y)S_{1}-yS_{2}}{z\left(S_{1}+S_{2}\right)}$$

where, for brevity, we introduced new symbols s_1_ and s_2_, defined as follows:

$$s_{1}=\sqrt{x^{2}+y^{2}+z{\left(x,y\right)}^{2}},s_{2}=\sqrt{{\left(x_{1}^{\prime }+\frac{\mathrm{atan}\left(\frac{-x}{\sqrt{y^{2}+z^{2}}}\right)}{\beta_{max}}\left(x_{1}^{\prime }-x_{2}^{\prime }\right)-x\right)}^{2}+{\left(y_{1}{}^{\prime }-y\right)}^{2}+{\left(z\left(x,y\right)\right)}^{2}}$$

A direct determination of the surface *z*(*x*, *y*) from these equations is not possible, due to the nonlinear entanglements of *z* appearing at both sides of Equation (8) and Equation (9) and the integrability condition violation [12,13,14,15,16]. For this reason, a different approach was used. First, we divided Equation (8) by Equation (9), which allowed identical denominators to be eliminated. Thus, we arrived at the following formula:

(10) $$\frac{\partial z}{\partial x}\left[(y_{1}{}^{\prime }-y)S_{1}-yS_{2}\right]=\frac{\partial z}{\partial y}\left[\left(x_{1}^{\prime }+\frac{\mathrm{atan}\left(\frac{-x}{\sqrt{y^{2}+z^{2}}}\right)}{\beta_{max}}\left(x_{1}^{\prime }-x_{2}^{\prime }\right)-x\right)S_{1}-xS_{2}\right]$$

This allows the expression describing the ratio of two partial derivatives, *z_x_* and *z_y_*, to be obtained, which will be denoted as *ξ*. Accordingly, the following formula can be obtained and considered as the merit function of the searched surface geometry:

(11) $$\delta =\xi \left(x,y,z\left(x,y\right)\right)-\frac{\left[\left(x_{1}^{\prime }+\frac{\mathrm{atan}\left(\frac{-x}{\sqrt{y^{2}+z^{2}}}\right)}{\beta_{max}}\left(x_{1}^{\prime }-x_{2}^{\prime }\right)-x\right)S_{1}-xS_{2}\right]}{\left[(y_{1}{}^{\prime }-y)S_{1}-yS_{2}\right]}$$

Now, we search for such a surface *z*(*x*, *y*), which minimizes *δ*. Generally, without any idea of the possible solutions, this would be hard to achieve. However, in our case, it is possible to deduce the initial candidate from the optical rules. Specifically, one can notice that, assuming *x*_1_’ = *x*_2_’, the searched surface corresponds exactly to the shape of a slanted ellipsoid, with the foci located at points (0, 0, 0) and (*x*_1_’, *y*_1_’, 0). The idea of the algorithm was to gradually move apart points (*x*_1_’, *y*_1_’, 0) from (*x*_2_’, *y*_1_’, 0) and, accordingly, correct the *z*(*x*, *y*) surface to minimize *δ*. The correction was performed in loops by searching the optimum coefficients of the so-called corrective polynomial, which is defined as follows:

(12) $$\varepsilon \left(x,y\right)=a_{00}+a_{10}\left(x-x_{0}\right)+a_{01}\left(y-y_{0}\right)+a_{11}\left(x-x_{0}\right)\left(y-y_{0}\right)+\;a_{21}{\left(x-x_{0}\right)}^{2}\left(y-y_{0}\right)+a_{IJ}{\left(x-x_{0}\right)}^{I}{\left(y-y_{0}\right)}^{J}$$

In our optimization approach, the idea of an orthogonal descent minimization scheme was applied in the space stretched over (*x*_0_, *y*_0_, *a_ij_*). The multi-dimensional gradient of the following form was calculated:

(13) $$\nabla \delta =\left[\frac{\partial \delta }{\partial x_{0}},\frac{\partial \delta }{\partial y_{0}},\frac{\partial \delta }{\partial a_{0}},\frac{\partial \delta }{\partial a_{00}},\frac{\partial \delta }{\partial a_{01}},\frac{\partial \delta }{\partial a_{11}},\ldots ,\frac{\partial \delta }{\partial a_{IJ}}\right]$$

Then, the polynomial coefficients were changed into loops, according to the “direction” determined by ∇δ, namely:

(14) $${\left[\begin{array}{c} x_{0}\\ y_{0}\\ a_{0}\\ a_{00}\\ a_{01}\\ \ldots \\ \ldots \end{array}\right]}_{\left(n+1\right)}={\left[\begin{array}{c} x_{0}\\ y_{0}\\ a_{0}\\ a_{00}\\ a_{01}\\ \ldots \\ \ldots \end{array}\right]}_{\left(n\right)}-\mu {\left[\begin{array}{c} \nabla \delta^{\left(1\right)}\\ \nabla \delta^{\left(2\right)}\\ \nabla \delta^{\left(3\right)}\\ \nabla \delta^{\left(4\right)}\\ \nabla \delta^{\left(5\right)}\\ \ldots \\ \ldots \end{array}\right]}_{\left(n\right)}$$

where *μ* is the algorithm optimization variable, $\nabla \delta^{\left(i\right)}$ corresponds to the *i*-th component of ∇δ, as presented in Equation (13), and *n* and *n* + 1 represent the consecutive loop numbers. Fortunately, the merit function appeared to not have many local minima, so the algorithm converged efficiently. This was manifested by the fast decrease in the ∇δ components. Typically, we ordered the algorithm to stop when they dropped below 0.1% of the initial levels. It is important that the designed surface meets the integrability condition, which guarantees surface continuity and smoothness (also, the first-order spatial derivatives are continuous). From a technological perspective, such a mirror can be relatively easily fabricated.

## 3. Results

**Figure 9:**
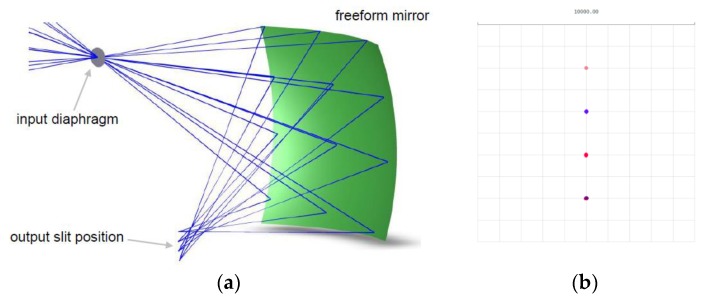
(**a**) Screenshot of the Optic Studio implementation using 3D visualization. (**b**) Spot diagram. The mirror was designed with the following inputs: *z*(0, 0) = 5 cm, d*x* = d*y* = 10 μm, *x*_1_’ = −5 cm, *x*_2_’ = −4.4 cm, *y*_1_’ = 2 cm, *β*_max_ = 60°, and *α*_max_ = 45°.

The discussed method was implemented numerically. The algorithm requires the following inputs: *z*(0, 0), d*x*, d*y*, *x*_1_’, *x*_2_’, *β*_max_, and *α*_max_. Alternatively, instead of *β*_max_ and *α*_max_, one can define the projected size of the mirror by specifying *x*_max_ and *y*_max_. The calculation time is short; it is a matter of single minutes in the case of 1 inch (2.54 cm) optics designed at a 10 μm spatial resolution (CPU 3.5 GHz, 16 GB RAM). The obtained surfaces were exported in the form of grid sag files in Optic Studio [17] for performance verification via ray-tracing. An example of such a simulation is presented in Figure 9. 

The presented visualization and corresponding spot diagram were obtained for several representative fields, both boundary and intermediate (Table 1). 

**Figure 10:**
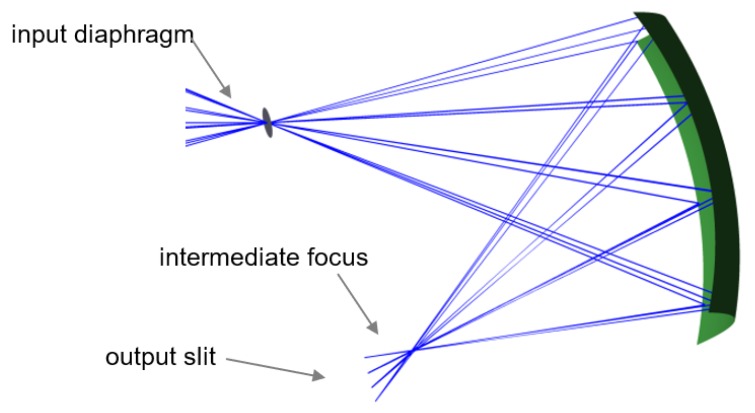
Side view of the setup, showing a tight intermediate focus.

The obtained results prove that there exists a linear relationship between the spatial location of the rays in the plane of the sensor (image plane) and angle *β*, irrespective of angle *α*. This property was the fundamental goal to be achieved. Additionally, the creation of the desired sharp intermediate focus could be observed (Figure 10).

**Figure 11:**
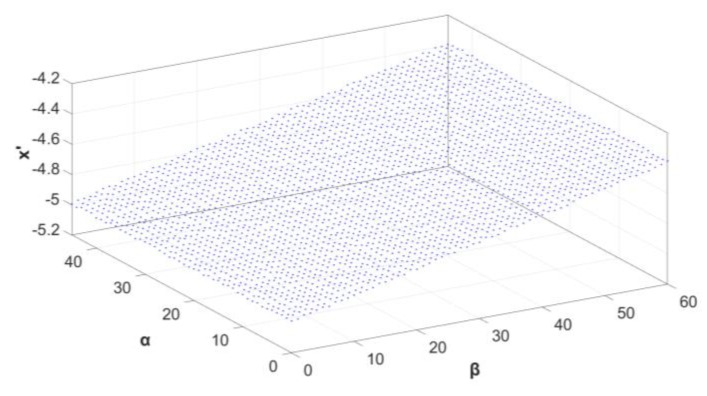
Results of the sensor’s simulated response.

We also developed a quantitative performance prediction analysis of the sensor for the whole range of incoming directions (0:α_max_; 0:β_max_). For each discrete {*α*, *β*} pair, ray tracing, which was realized in Optic Studio, enabled *x*’, the *x*-coordinate of the spot in the image (sensor) plane, to be calculated. This value corresponds to the activated pixel number, which is used in the sensor for the angle evaluation (Figure 11). 

Again, a satisfactory linear relationship between *x*’ and *β* was observed, with practically no impact of the *α* angle. This resulted in a constant 0.1 mm/° angular resolution of the sensor in a full field of view. The calculated root-mean-square error of *x*’ equaled about 0.015 mm, corresponding to 0.15°, a level satisfactory in most practical applications. Regarding the resistance of the designed solution against potential technological misalignments, it is fortunately not a critical issue. It was confirmed by numerical tolerancing of the case study AOI sensor. Here, the optical system performs a non-imaging task, where a short equivalent-focus freeform mirror cooperates with relatively large photosensitive pixels (typically PIN-photodiodes). 

## 4. Discussion

The paper presents a new approach to the design of AOI sensors. The novelty results from the application of a freeform mirror and the method of how to design such a component. In comparison to the existing solutions, the proposed architecture of the sensor enables the following to be obtained:

- A linear response to the measured angle (constant angular resolution) in the full range;
- Reduced stray light;
- A higher dynamic range; and
- An increased compactness.

The only optical component of the sensor is a freeform mirror, which can be designed for specific purposes (angular range, resolution, and sensor size) by applying the method, which has been described precisely in the paper. The geometry of this mirror solves the problem of a non-linear transformation between AOI and the position of a light pencil on a linear photo-detector array. Such a property in optics is associated with f-theta lenses [18]. However, the presented solution offers two additional assets: The creation of an intermediate focus and shifting in the destination of rays (output slit or location of the detector) from the incidence plane. Both of these features, together with the small circular entrance aperture, enable the stray light in the sensor to be dramatically reduced, if compared to standard solutions, where light enters the sensor through significantly larger diaphragms. From an optical perspective, the designed reflective freeform surface performs like an f-theta lens in one cross-section and a perfect lens, combined with an angle-deviating prism in the second (orthogonal) one. The design methodology is based on 3D geometrical optics laws and differential calculus, so it is appropriate for configurations where diffraction effects do not play a significant role (the diaphragm is much larger than wavelengths of light). It should be underlined, however, that in the case of very-powerful-laser detection, diffraction on the diaphragm edge can produce a sufficient amount of stray light to activate inappropriate pixels. In addition, the non-zero thickness of the diaphragm generates standard geometrical reflections from its edge, which adds another contribution to the unwanted stray light. The importance of the intermediate focus is proved by the fact that it brings an efficient remedy for both factors. Due to the elimination of stray light issues, the sensor can reach a dynamic range limited purely by the photo-detection process, and thus, apart from high-power sources (LWR/LWS), it may also be useful for low-light applications (for example, star trackers).

## Figures and Tables

**Table 1:** Field angles used in the simulation presented in Figure 9.

α	0	22	45	0	22	45	0	22	45	0	22	45
β	0	0	0	20	20	20	40	40	40	60	60	60
